# MODIT: MOtif DIscovery in Temporal Networks

**DOI:** 10.3389/fdata.2021.806014

**Published:** 2022-02-23

**Authors:** Roberto Grasso, Giovanni Micale, Alfredo Ferro, Alfredo Pulvirenti

**Affiliations:** ^1^Department of Physics and Astronomy, University of Catania, Catania, Italy; ^2^Department of Clinical and Experimental Medicine, University of Catania, Catania, Italy

**Keywords:** temporal networks, network motifs, motif search algorithms, motif counting, network analysis, data mining

## Abstract

Temporal networks are graphs where each edge is linked with a timestamp, denoting when an interaction between two nodes happens. According to the most recently proposed definitions of the problem, motif search in temporal networks consists in finding and counting all connected temporal graphs *Q* (called motifs) occurring in a larger temporal network *T*, such that matched target edges follow the same chronological order imposed by edges in *Q*. In the last few years, several algorithms have been proposed to solve motif search, but most of them are limited to very small or specific motifs due to the computational complexity of the problem. In this paper, we present MODIT (MOtif DIscovery in Temporal Networks), an algorithm for counting motifs of any size in temporal networks, inspired by a very recent algorithm for subgraph isomorphism in temporal networks, called TemporalRI. Experiments show that for big motifs (more than 3 nodes and 3 edges) MODIT can efficiently retrieve them in reasonable time (up to few hours) in many networks of medium and large size and outperforms state-of-the art algorithms.

## 1. Introduction and Related Works

Networks (also named graphs) are tools for the description and analysis of entities, called nodes, that interact with each other by means of edges. There are many types of data that can be represented by graphs, including computer networks, social networks, communication networks, biological networks, and so on. A wide range of domains can be modeled and studied with static networks but many complex systems are fully dynamic, indeed interactions between entities change over time. Systems of this type can be modeled as *temporal networks*, in which edges between nodes are associated with temporal information such as, for example, the duration of the interaction and the instant in which the interaction begins. Annotations of edges with temporal data is important to understand the formation and the evolution of such systems.

In literature, several definitions of temporal networks have been proposed (Holme and Saramaki, 2012; Masuda and Lambiotte, 2020). In few works, these are also referenced as dynamic (Carley et al., 2007), evolutionary (Aggarwal and Subbian, 2014) or time-varying (Casteigts et al., 2011) networks. In this paper, we define temporal network as a multigraph (i.e a graph where two nodes may interact multiple times). Each edge is associated with an integer, called timestamp, which denotes when two nodes interact.

In the last few years, there has been a growing interest in analyzing temporal networks and studying their properties. Analysis of temporal networks includes network centrality (Lv et al., 2019; Tsalouchidou et al., 2020), network clustering (Crawford and Milenkovic, 2018), community detection (Rossetti and Cazabet, 2018), link prediction (Divakaran and Mohan, 2020), graph mining (Sun et al., 2019), graph embedding (Torricelli et al., 2020), network sampling (Rocha et al., 2017), random models (Petit et al., 2018; Hiraoka et al., 2020; Singh and Cherifi, 2020), and epidemic spreading (Tizzani et al., 2018; Williams et al., 2019; Masuda and Holme, 2020). Extensive reviews of temporal networks and their main features can be found in Holme and Saramaki (2012, 2019), Masuda and Lambiotte (2020).

**Figure 1:**
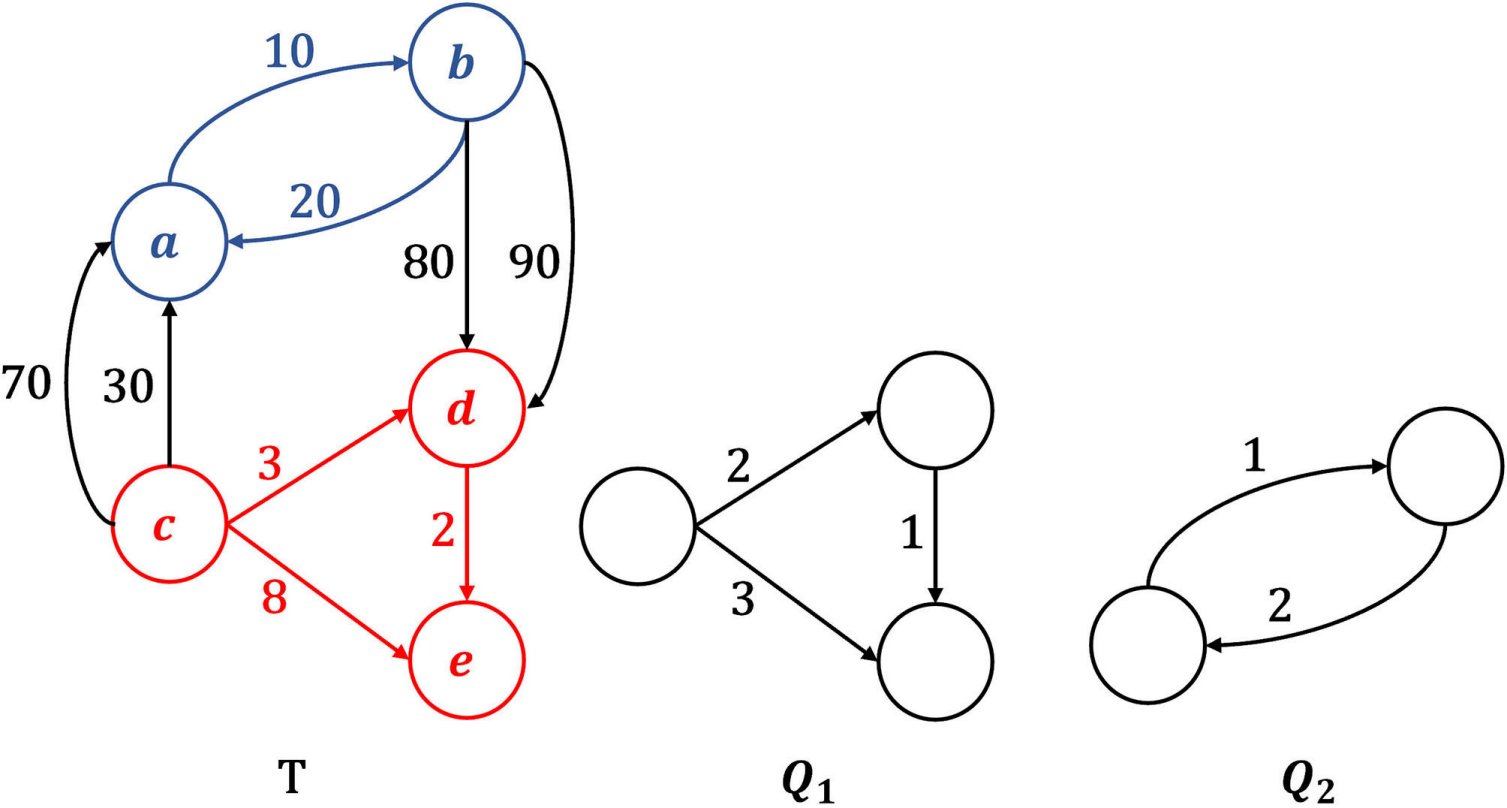
Example of motif Δ-occurrence in a temporal graph *T*, given Δ = 6. Motif **Q**_**1**_ has exactly one Δ-occurrence in **T**, which is the subgraph formed by nodes and edges colored in red. Motif **Q**_**2**_, instead, does not Δ-occur in *T*. In fact, the subgraph with blue nodes and blue edges is isomorphic to **Q**_2_ and respects the chronological order imposed by *Q*_2_'s edges, but its edges are not observed within the time window Δ.

In this work, we focus on motif search in temporal networks. Different definitions of temporal motifs have been proposed so far (Kovanen et al., 2011; Hulovatyy et al., 2015; Paranjape et al., 2017). Here, we follow the most recent definition proposed by Paranjape et al. (2017), which is becoming the most accepted one. A temporal motif is a temporal network where edges denote a succession of events. In addition to the original definition proposed by Paranjape et al. (2017), simultaneous events, represented by edges with equal timestamps, are allowed, provided that such edges do not link the same pair of nodes. Temporal graphs *Q*_1_ and *Q*_2_ of Figure 1 are two examples of motifs. Applications of Temporal Motif Search include the creation of evolution rules that govern the way the network changes over time (Berlingerio et al., 2009; Ugander et al., 2013) allowing also to identify all the time an edge participates to particular pattern in a time window. A second application consists in the identification of motifs in temporal network at different time resolution to identify patterns at different time scale. Another application consists in temporal network classification using a feature representation based on the temporal motifs distribution (Tu et al., 2018).

Given a time interval Δ, we say that a motif *Q* Δ-occurs in *T*, iff: (i) *Q* is isomorphic (i.e., structurally equivalent) to a subgraph *S* of *T* (called an occurrence of *Q* in *T*), (ii) edges in *S* follow the same chronological order imposed by corresponding matched edges in *Q*, (iii) all interactions in *S* are observed in a time interval less than or equal to Δ (i.e., they are likely to be related each other). In Figure 1, motif *Q*_1_ Δ-occurs (Δ = 6 in the example) in *T*, while *Q*_2_ does not.

**Figure 2:**
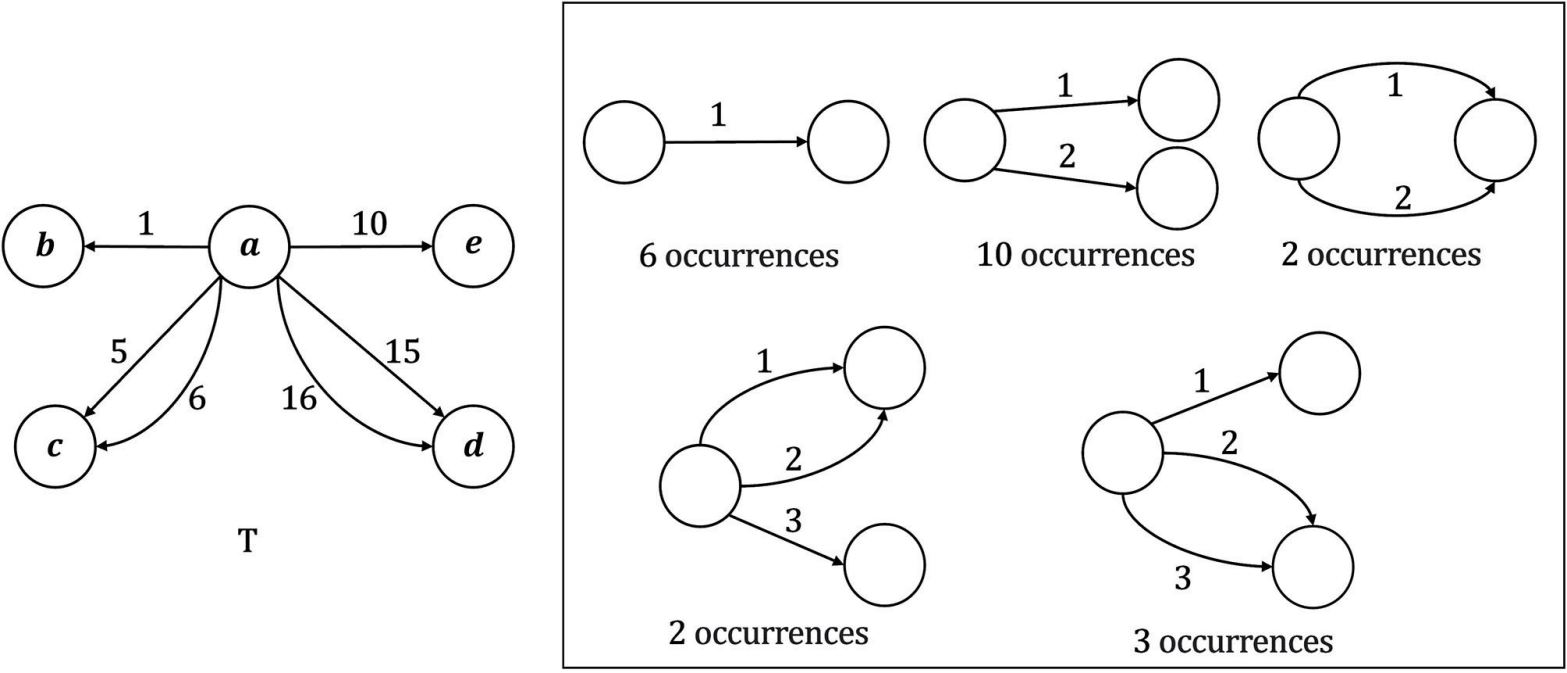
Example of application of the Temporal Motif Search (TMS) problem for a temporal graph **T**, given Δ = 10, *k* = 3, and *l* = 3. For each motif, the relative number of Δ-occurrences in *T* is reported.

For a given temporal graph *T* and time interval Δ, motif search aims at retrieving all motifs that Δ-occurs in *T*. In addition, for each such motif *Q*, we also count the number of occurrences of *Q* in *T*. It has been shown that Temporal Motif Search (TMS) problem is NP-complete, even for star topologies (Liu et al., 2019). For this reason, motif search is usually restricted to motifs with up to a certain number of nodes and edges. Given Δ = 10, Figure 2 shows all temporal motifs with up to 3 nodes and 3 edges that Δ-occur in a toy temporal graph, together with the corresponding number of occurrences.

Recently, several TMS algorithms have been introduced (Kovanen et al., 2011; Hulovatyy et al., 2015; Paranjape et al., 2017; Liu et al., 2019). However, the proposed solutions are limited to very small motifs or specific topologies.

Temporal motifs have been introduced for the first time by Kovanen et al. (2011). Authors define a motif as an ordered set of edges such that: (i) the difference between the timestamps of two consecutive edges must be less than or equal to a certain threshold Δ and (ii) if a node is part of a motif, then all its adjacent edges have to be consecutive (consecutive edge restriction).

In Hulovatyy et al. (2015) the consecutive edge restriction was relaxed and the authors considered only induced subgraphs, called graphlets, in order to reduce the computational complexity while obtaining approximate results.

Paranjape et al. (2017) describes a temporal motif as a sequence of edges ordered by increasing timestamps. More precisely, the authors define a *k*-node, *l*-edge, Δ-temporal motif as a sequence of *l* edges, *M* = (*u*_1_, *v*_1_, *t*_1_), (*u*_2_, *v*_2_, *t*_2_), …, (*u*_*l*_, *v*_*l*_, *t*_*l*_) that are time-ordered within a Δ duration, i.e., *t*_1_ < *t*_2_ ⋯ < *t*_*l*_ and *t*_*l*_ − *t*_1_ ≤ Δ, such that the static graph induced by the edges is connected and has *k* nodes. The authors present an algorithm to efficiently calculate the frequencies of all possible directed temporal motifs with 3 edges. For bigger motifs they use a naive algorithm that first computes static matches, then filters out occurrences which do not match the temporal constraints.

To tackle with the NP-completeness of TMS, approximate solutions have been proposed too. Liu et al. (2019) propose a general sampling framework to estimate motif counts. It consists in partitioning time into intervals, finding exact counts of motifs in each interval and weighting counts to get the final estimate, using importance sampling.

In this paper, we present a new motif search algorithm, called MODIT (MOtif DIscovery in Temporal networks). The method is inspired by the temporal subgraph matching algorithm TemporalRI (Locicero et al., 2021; Micale et al., 2021). Our algorithm overcomes many of the limitations imposed by other motif search methods. In fact, MODIT is general and can search for motifs of any size. It has no consecutive edge restriction and allows edges with equal timestamps, provided that they do not link the same pair of nodes.

The rest of the paper is organized as follows. In section 2, we give preliminary definitions about temporal networks and temporal motif search, then we illustrate MODIT and evaluate its computational complexity. In section 3, we assess the performance of MODIT on a dataset of real networks and compare it with the algorithm presented in Paranjape et al. (2017). Finally, section 4 ends the paper.

## 2. Methods

### 2.1. Preliminary Definitions

In this section, we formally define the concepts of temporal graph and temporal motif, then we introduce the temporal motif search problem.

#### 2.1.1. Temporal Graph

A *temporal graph* (or network) *G* is a pair of sets (*V, E*), where *V* is the set of nodes and *E* ⊆ *V* × *V* × ℝ is the set of edges. Each edge is a triple (*s, d, t*) where *s* is the source node, *d* is the destination node and *t* is the timestamp, denoting the moment or the time interval in which the two nodes interact.

By definition, a temporal graph is a multigraph, because there can be multiple edges between two nodes. However, triplets in *E* are distinct, therefore such edges need to have different timestamps. With *e*.*source*, *e*.*dest*, and *e*.*time* we denote the source, the destination and the timestamp of edge *e*, respectively. A temporal graph *G* = (*V, E*) is *undirected* if ∀(*s, d, t*) ∈ *E*(*G*) we have (*d, s, t*) ∈ *E*(*G*), otherwise it is *directed*. With *Inc*(*v*) we denote the set of all edges that are incident to node *v*, i.e., having *v* as source or destination.

#### 2.1.2. Temporal Motif

Let *Q* = (*V, E*) a connected temporal graph with *l* edges and (*t*_1_, *t*_2_, …, *t*_*l*_) the sequence of *Q*'s edges timestamps in ascending order. *Q* is a *temporal motif* iff: (i) *t*_1_ = 1, (ii) *t*_*i*+1_ − *t*_*i*_ ≤ 1∀1 ≤ *i* ≤ *l* − 1.

In other words, a temporal motif *Q* can be considered a sort of standardized temporal graph, in which edge timestamps denote an order in which events happen starting from the initial event (i.e., event 1). Edges with equal timestamps (if any) in *Q* represent simultaneous event. Examples of temporal motifs are the graphs *Q*_1_ and *Q*_2_ depicted in Figure 1.

To establish if a temporal graph contains a temporal motif, we need to introduce the concept of Temporal Subgraph Isomorphism. We follow the definition reported in Micale et al. (2021).

#### 2.1.3. Temporal Subgraph Isomorphism

Given two temporal graphs *Q* = (*V*_*Q*_, *E*_*Q*_) and *T* = (*V*_*T*_, *E*_*T*_), called, respectively, *query* and *target*, and an integer Δ, the *Temporal Subgraph Isomorphism* (TSI) problem consists in finding an injective function *f*:*V*_*Q*_ → *V*_*T*_, called *node mapping*, and an injective function *g*:*E*_*Q*_ → *E*_*T*_, called *edge mapping*, such that the following conditions hold:

1. ∀*e*_*Q*_ = (*u, v, t*_*Q*_) ∈ *E*_*Q*_ ∃ *e*_*T*_ ∈ *E*_*T*_ s.t. *e*_*T*_ = *g*(*e*_*Q*_) = (*f*(*u*), *f*(*v*), *t*_*T*_);
2. $\forall e_{Q},e_{Q}^{\prime }\in E_{Q}$ s.t. $e_{Q}.time\leq e_{Q}^{\prime }.time\Rightarrow g\left(e_{Q}\right).time\leq g\left(e_{Q}^{\prime }\right).time$;
3. $\forall e_{Q},e_{Q}^{\prime }\in E_{Q}$ $\left|g\left(e_{Q}\right).time-g\left(e_{Q}^{\prime }\right).time\right|\leq \Delta$.

The first condition ensures that the edge mapping is consistent with the node mapping. The second condition requires that the chronological order between query edges is respected in the target network. The third condition imposes that all matching target edges are observed within a fixed time interval Δ.

The TSI problem can have one or more solutions. In this case, we say that *Q* Δ*-occurs* in *T*. Given an edge mapping *g* between *Q* and *T*, a Δ*-occurrence* of *Q* in *T* is a temporal graph *S* formed by edges *g*(*q*_1_), *g*(*q*_2_), …, *g*(*q*_*k*_) and all nodes that are sources or destinations of at least one of these edges.

In Figure 1, given Δ = 6, query *Q*_1_ Δ-occurs in target *T* and the corresponding occurrence is the subgraph of *T* highlighted in red. Query *Q*_2_, instead, has no Δ-occurrences in *T*. Indeed, there is only one subgraph of *T* (highlighted in blue) that is isomorphic to *Q*_2_ but violates the Δ constraint on edge timestamps.

Finally, we define the temporal motif search problem.

#### 2.1.4. Temporal Motif Search

Given a temporal graph *T* = (*V*_*T*_, *E*_*T*_) and three integers *k*, *l* and Δ, the Temporal Motif Search (TMS) problem consists in: (i) retrieving all temporal motifs that Δ-occurs in *T* and have at most *k* nodes and *l* edges, (ii) counting the number of Δ-occurrences of such motifs.

An example of application of the TMS problem is shown in Figure 2 where *k* = 3, *l* = 3 and Δ = 10.

### 2.2. The MODIT Algorithm

In what follows we introduce a new algorithm for solving the TMS problem, called MODIT (MOtif DIscovery in Temporal networks).

Given three parameters *k*, *l*, and Δ, MODIT scans a temporal graph *T* to retrieve all temporal motifs with at most *k* nodes and *l* edges which Δ-occur in *T* and counts the number of Δ-occurrences of each motif in *T*.

For each newly identified occurrence, the algorithm performs the following steps:

1. Standardization of edge timestamps;
2. Construction of the canonical form and identification of the corresponding temporal motif;
3. Update of the count of the number of motif occurrences.

The search starts from the smallest motif occurrences formed by single edges. We call these edges seeds. Each of these occurrences is then recursively extended by adding one edge at the time until the specified maximum number of nodes and edges is reached.

MODIT can work on both undirected and directed graphs. For ease of presentation, we illustrate the functioning of the algorithm for undirected networks. However, all the procedures presented here can be easily adapted to directed networks.

The pseudocode of MODIT is reported in Algorithm 1. All motifs retrieved by the algorithm, together with the number of their occurrences, are stored in a hash map *motifMap*, empty at the beginning (line 1). Each motif in the map is uniquely represented by a string, called canonical form. In addition, since the same occurrence of a motif may be examined multiple times, we also need to store all the distinct retrieved subgraph occurrences in a hash set *minedSubgraphs* (line 2). For each edge *e* = (*u, v, t*) of the target graph, MODIT performs the following steps. First, *e* and its nodes are embedded in a new occurrence graph *S*, which is added to the set *minedSubgraphs* (lines 4–7). The timestamp of the seed edge *e* is stored in a variable *minTime*, which will be used throughout the search to avoid scanning some subgraphs of *T* multiple times (line 8). Each edge that will be added to the subgraph must have a timestamp greater than or equal to *minTime*. To ensure that each subgraph obtained by expanding *S* does not violate the Δ temporal constrain, MODIT also uses three auxiliary variables: *timestampSet*, *maxTime*, and λ (lines 9–12). Variable *timestampSet* is a multi-set containing the timestamps of the currently examined subgraph *S* (at the beginning just *t*). *maxTime* will store at each step the maximum timestamp of edges in *S*. λ represents how much we can extend the time window covered by edges in *S* (i.e., the difference between the maximum and the minimum timestamps), without exceeding Δ. *maxTime* and λ are initialized and kept updated during the search using Algorithm 2 (line 13).

**Algorithm 1:** MODIT(*G*, Δ, *k*, *l*).

1	Let *motifMap* be an empty hash map.
2	*minedSubgraphs* = ∅
3	**for each** *e* = (*u, v, t*) ∈ *E*(*G*) **do**
4	Let *S* be an empty graph.
5	*V*(*S*) = *V*(*S*) ∪ {*v*}
6	*E*(*S*) = *E*(*S*) ∪ {*e*}
7	*minedSubgraphs* = *minedSubgraphs* ∪ *S*
8	*minTime* = *t*
9	*maxTime* = 0
10	λ = + ∞
11	Let *timestampSet* be an empty multiset
12	*timestampSet* = *timestampSet* ∪ {*t*}
13	UpdateBounds (*timestampSet*, Δ, *minTime, maxTime*, λ)
14	*M* = StandardizeTimestamps (*S*)
15	*C* = ComputeCanonization (*M*)
16	UpdateOccurrences (*motifMap, C*)
17	RecursiveSearch (*S, u*, Δ, *timestampSet, minTime*, *maxTime*, λ, *motifMap, k, l, minedSubgraphs*)
18	RecursiveSearch (*S, v*, Δ, *timestampSet, minTime*, *maxTime*, λ, *motifMap, k, l, minedSubgraphs*)
19	**return** *motifMap*

**Algorithm 2:** UpdateBounds(*timestampSet*, Δ, *minTime, maxTime*, λ).

1	*maxTime* = MAX (*timestampSet*)
2	λ = Δ − *maxTime* + *minTime*
3	**return**

Next, the timestamps of the current subgraph *S* are standardized, i.e they are modified in order to transform *S* into a temporal motif *M*, in compliance with the definition provided in section 2.1 (line 14). Standardization of timestamps aims at identifying the motif *M* of which *S* is an occurrence and works as follows. First, the list of edge timestamps in *S* (without duplicates) is sorted in ascending order. Then, each edge of *S* is assigned the rank of the corresponding timestamp in the sorted list.

**Figure 3:**
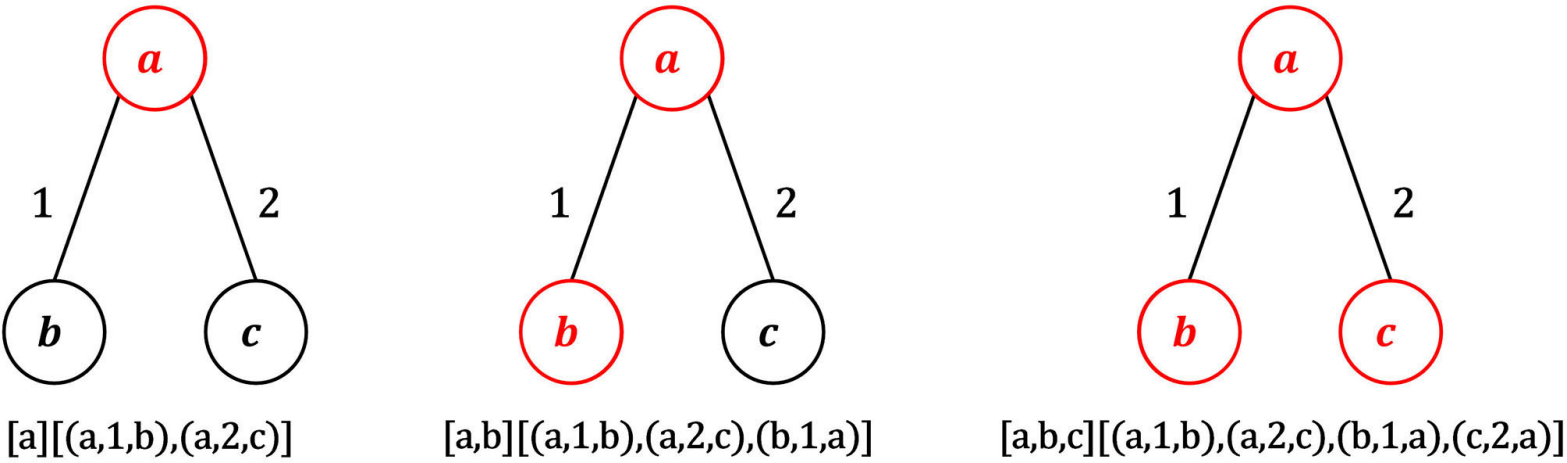
Example of construction of the canonical form. Node *a* has the highest degree (2), so *a* is the first node of the ordering. Nodes *b* and *c* have the same degree, so we need to examine the adjacency lists of *b* and *c* sorted by timestamp and destination node. The first edge in the sorted list of *b* has timestamp 1, while the first edge in the sorted list of *c* has timestamp 2, so the second node of the ordering is *b* and the third one is *c*. Following such node ordering, the canonical form of the graph in the figure is {(*a*, 1, *b*), (*a*, 2, *c*), (*b*, 1, *a*), (*c*, 2, *a*)}.

Standardization alone may produce distinct motifs that are actually structurally equivalent and in which equivalent edges have the same timestamps. To avoid this, a canonical form *C* is extracted from *M* (line 15). The canonical form is a string that uniquely represents a motif, so that two motifs that are equivalent have exactly the same canonical forms. *C* is obtained by concatenating motif edges based on a given order of motif nodes. Nodes are first ordered according to their degree. Possible ties are solved comparing their sorted adjacency lists, in which edges are ordered by timestamp and, in case of ties, by destination node. Following the calculated node ordering, sorted adjacency lists of nodes are concatenated to yield the canonical form. Figure 3 shows an example of computation of canonical form. In the depicted motif, node *a* is the first node in the ordering, since it has the maximum degree (2). Nodes *b* and *c* have both degree 1. If the adjacency lists of *b* and *c* are sorted by timestamps, *b* comes before *c* because the first edge in the sorted adjacency list of *b* has timestamp 1, while the first edge in the sorted adjacency list of *c* has timestamp 2. Following the node ordering {*a, b, c*}, sorted adjacency lists of *a*, *b* and *c* are appended to yield the final canonical form of the motif, i.e., the string *C* = {(*a*, 1, *b*), (*a*, 2, *c*), (*b*, 1, *a*), (*c*, 2, *a*)}.

After constructing the canonical form, the number of occurrences of *M* is incremented in *motifMap* (line 17).

Next, MODIT continues the search of new occurrences by extending *S* edge by edge, starting from an anchor node. This is done using the recursive procedure RecursiveSearch described in Algorithm 3 (lines 17-18). The first two calls to the procedure will extend the seed using both endpoints as anchors. In general, *S* will be extended by adding an edge which is not already present in *S* and is incident to an anchor node already present in *S*.

**Algorithm 3:** RecursiveSearch(*S*, *a*, Δ, *timestampSet*, *minTime*, *maxTime*, λ, *m*, *k*, *l*, *minedSubgraphs*).

1	**if** |*E*(*S*)| ≥ *l* **then**
2	**return**
3	**for each** *e* = (*u, a, t*) ∈ *Inc*(*v*) ∧ *e* ∉ *E* (*S*) **do**
4	**if** *t* ∈ [*minTime, maxTime* + λ] **then**
5	**if** *u* ∉ *V*(*S*) **then**
6	**if** |*V* (*S*)| ≤ (*k* − 1) **then**
7	*V*(*S*) = *V*(*S*) ∪ {*u*}
8	*added* = *true*
9	**else**
10	**continue**
11	*E*(*S*) = *E*(*S*) ∪ {*e*}
12	**if** *S* ∉ *minedSubgraphs* **then**
13	*timestampSet* = *timestampSet* ∪ {*t*}
14	*minedSubgraphs* = *minedSubgraphs*∪*S*
15	UpdateBounds (*timestampSet*, Δ, *minTime*, *maxTime*, λ)
16	*M* = StandardizeTimestamps (*S*)
17	*C* = ComputeCanonization (*M*)
18	UpdateOccurrences (*m, canonicalForm*)
19	RecursiveSearch (*S, u*, Δ, *timestampSet, minTime*, *maxTime*, λ, *m, k, l, minedSubgraphs*)
20	RecursiveSearch (*S, a*, Δ, *timestampSet, minTime*, *maxTime*, λ, *m, k, l, minedSubgraphs*)
21	*timestampSet* = *timestampSet*\{*e*.*t*}
22	*E*(*S*) = *E*(*S*)\{*e*}
23	**if** *added* = *true* **then**
24	*V*(*S*) = *V*(*S*)\{*u*}
25	*added* = *false*
26	UpdateBounds (*timestampSet*, Δ, *minTime, maxTime*, λ)
27	**return**

The structure of RecursiveSearch procedure is very similar to Algorithm 1. First, we check if the currently examined subgraph has reached the maximum allowed number of edges (lines 1-2). If so, the recursive algorithm stops, otherwise the search goes on, considering all possible edges *e* = (*u, a, t*) not already present in *S* and incident to the anchor node *a* (line 3). For each such edge, MODIT ensures that by adding *e* to *S*, the Δ temporal constrain is not violated (line 4). Based on the current values of *minTime*, *maxTime* and λ, we can add *e* to *S* without violating the Δ constraint iff *minTime* ≤ *t* ≤ *maxTime* + λ. We impose that *t* is no lower than *minTime*, i.e., the timestamp of the seed edge, to reduce the number of redundant candidates generated.

If *e* does not violate the Δ constraint, before adding it to *S* (line 11), we do the following steps. First, we check if *e* does not connect two nodes already present in *S* (line 5). If so, we verify if the currently examined subgraph has not reached the maximum allowed number of nodes (line 6). In this case, node *u* is added to *S* (line 7). Otherwise, we proceed with the next edge (line 10).

Boolean conditions expressed in line 4 does not prevent examining some subgraphs multiple times. Therefore, before going on with the search, we need to check if *S* has not been already examined before (line 12). This is done by simply comparing the list of edge ids of *S* and each subgraph of the *minedSubgraphs* set.

If *S* is new, we follow the same steps performed in Algorithm 1. First, we include *t* in *timestampSet* and add *S* to *minedSubgraph* (lines 13–14). We update temporal auxiliary variables *minTime*, *maxTime* and λ (line 15). Then, edge timestamps are standardized to obtain a temporal motif *M* (line 16). From *M* we extract the canonical form *C* (line 17) and increase the number of its occurrences (line 18).

Next, Algorithm 3 is called recursively twice using the endpoints of *u* as anchor nodes (lines 19–20). After returning from the recursive calls, backtracking is performed (lines 21–26). Backtracking implies: (i) removing from *S* the last added edge *e*, (ii) removing from *S* the last added node, (iii) removing the timestamp of *e* from *timestampSet*, (iv) updating auxiliary variables *maxTime* and λ.

### 2.3. MODIT Complexity Analysis

In this subsection, we analyze the complexity of MODIT. The search starts from the smallest motif occurrences formed by single edges. Therefore, the for-loop in Algorithm 1 is performed |*E*(*G*)| times, where *G* is the target graph. Inside the loop, MODIT tries to expand each occurrence as long as possible. Lines 4–7 of Algorithm 3 require constant time because they are applied to a subgraph formed by only one edge.

Now let's analyze the complexity of Algorithm 3. Let *d*_*max*_ the maximum node degree of *G*. The for-loop in Algorithm 3 is performed, in the worst case, *d*_*max*_ times. Assuming Δ = ∞, all *d*_*max*_ edges are candidates to extend the motif. Lines 4–14 of Algorithm 3 require a constant time. The complexity of Algorithm 2 depends on the number of distinct timestamps of the current motif. In the worst case there are *l* edges and all timestamps are different. Identifying the minimum and maximum requires an ordering of timestamps that has linear complexity. The rest of the operations can be done in constant time. So, the complexity of Algorithm 2 is *O*(*l*). However, in practice, these operations are done faster because MODIT stores timestamps in a data structure that is self-sorted as elements are inserted/removed. Standardization of timestamps requires linear time with respect to the number of edges. Since a motif can have at most *l* edges, the complexity is *O*(*l*). The time required to build the canonical form depends on the number of nodes and the number of edges of the motif. Sorting the adjacency list of a node requires, in the worst case, *l* operations. Since a motif can have at most *k* nodes, sorting their adjacency lists requires at most *k* · *l* operations. The ordering of nodes has linear complexity with respect to the number of nodes, thus performs in the worst case, *k* operations. Therefore, the number of operations required to calculate the canonical form is at most *k* · *l*+*k*. Updating the number of occurrences of a motif is done using a hash map, so it takes constant time.

To derive the final complexity of Algorithm 3, we need to evaluate the maximum depth of the recursion. Each call to Algorithm 3 adds one edge at the time, so the maximum recursion depth is *l*. Assuming no early backtracking, this implies that the complexity of the recursive procedure is $O\left({\left(l\cdot k\cdot d_{max}\right)}^{l}\right)$. So overall, the complexity of MODIT is $O\left(|E\left(G\right)|\cdot {\left(l\cdot k\cdot d_{max}\right)}^{l}\right)$.

## 3. Results

MODIT has been implemented in Java and tested on two datasets of real temporal networks of different sizes, denoted as Dataset 1 and Dataset 2, respectively. Table 1 lists the main features of the networks of the two datasets. For each graph we report the number of nodes, the number of edges, the number of distinct timestamps and the resolution, i.e., the minimum difference between consecutive timestamps.

**Table 1:** Datasets of temporal networks used for the experiments.

**Dataset**	**Network**	**Nodes**	**Edges**	**Timestamps**	**Resolution**
Dataset 1	SFHH-Conf	403	70,261	3,509	20
	As-Topology	34,761	155,507	32,824	1
	Contacts-Dublin	10,972	415,912	76,944	20
	Enron-Email	86,978	1,134,990	213,218	1
	Digg-Friends	279,374	1,729,983	1,644,369	1
	Yahoo-Messages	100,001	3,157,315	898,174	1
Dataset 2	CollegeMsg	1,899	59,835	58,911	114,878
	Email-Eu-core-temporal-Dept1	309	61,046	35,097	21,799
	Email-Eu-core-temporal-Dept2	162	46,772	32,340	507
	Email-Eu-core-temporal-Dept3	89	12,216	8,911	2,635
	Email-Eu-core-temporal-Dept4	142	48,141	26,496	88
	Email-Eu-core-temporal	986	332,334	207,880	2,797

*For each network we indicate the number of nodes, the number of edges, the number of distinct timestamps and the resolution, i.e., the minimum difference between the timestamps of two consecutive edges*.

Dataset 1 is formed by six networks: SFHH-Conf, As-Topology, Contacts-Dublin, Enron-Email, Digg-Friends, and Yahoo-messages.

SFHH-Conf is a network that describes the interactions between the 405 participants of the SFHH conference in Nice, France (Génois and Barrat, 2018). As-Topology is a peer-to-peer communication network between autonomous systems, with data collected between February and March of 2010. Contacts-Dublin is a contact network of attendees at the *Infectious SocioPatterns* event held in the Science Gallery in Dublin, Ireland (Isella et al., 2011). Enron-Email is a network of e-mail exchanges of *Enron corporation* employees between 1999 and 2003 (Keila and Skillicorn, 2005). Digg-Friends describes friendly bonds between users of *Digg*, a web news aggregator used in America (Hogg and Lerman, 2012). It is based on data collected in 1 month of 2009. Yahoo-messages represents the exchange of e-mails between users of *Yahoo-Mail* in 1 month of 2010.

Dataset 2 includes 6 temporal networks taken from the SNAP dataset^1^. CollegeMsg consists of private messages sent on an online social network at the University of California, Irvine (Panzarasa et al., 2009). The network Email-Eu-core-temporal was generated using email data from a large European research institution. Only emails exchanged between institution members were taken into account. Email-Eu-core-temporal-Dept1, Email-Eu-core-temporal-Dept2, Email-Eu-core-temporal-Dept3 and Email-Eu-core-temporal-Dept4 are four sub-networks including communications between members of four different departments of the institution (Paranjape et al., 2017).

We first ran MODIT on each network of Dataset 1 for different combinations of values of Δ, *k* (maximum number of motif nodes) and *l* (maximum number of motif edges). Then, we compared MODIT to the algorithm proposed by Paranjape et al. (2017), which is included in the SNAP platform for network analysis and uses the same definition of temporal motif. All other methods were discarded because they use different definitions of temporal motifs. This comparison was done on the networks of Dataset 2.

All experiments were performed on an Intel Core i5-7500 processor with 16GB of RAM, 10 of which were used for the Java Virtual Machine.

### 3.1. Experiments on Dataset 1

In this section, we illustrate the results of the experiments on Dataset 1. We report in Tables 2–4 the results in terms of (i) execution times, (ii) number of distinct motifs identified, (iii) number of occurrences of the most frequent motif, and (iv) average number of motif occurrences. The experiments were performed for different combinations of values of Δ, *k* and *l*. Δ was set to *r*, 2*r*, and 3*r*, where *r* is the resolution of the temporal network. For *k* we used values 3, 4, and 5. *l* was varied as a function of *k* and set to values *k* − 1, 2 · (*k* − 1) and 3 · (*k* − 1). In some networks (in particular, in As-Topology and Enron-Email) and for some configurations of the parameters, MODIT went out of memory and was unable to finish the execution. In these cases we did not report any running time. This is due to the large number of distinct motifs present in the networks, which leads to an excessive growth of the map of motif counts, together with a large number of occurrences causing many recursive calls of Algorithm 3. In fact, as *k* and *l* increase, the number of motif topologies and the number of combinations of standardized timestamps increases exponentially [e.g., SFHH-conf network in the following configurations: (Δ = 3*r*, *k* = 3, *l* = 2), (Δ = 3*r*, *k* = 3, *l* = 4) and (Δ = 3*r*, *k* = 3, *l* = 6)]. This is confirmed by the high values of the number of occurrences of the most frequent motif and the average number of motif occurrences found for small values of *k* and *l*.

**Table 2:** Experiments on Dataset 1 with Δ = 3*r* and different combination of values of *k* and *l*.

**Configuration**	**Network**	**Time (*s*)**	**Mem (*GB*)**	**n**	**Max**	**AVG**
*k* = 3 *l* = 2	SFHH-Conf	3.06	<1	8	44,221	30,387
	As-Topology	83.10	5	10	16,068,985	1,885,655
	Contacts-Dublin	8.21	2	9	192,318	112,497
	Enron-Email	202.57	8	10	40,484,380	4,050,215
	Digg-Friends	48.16	2	9	259,097	37,211
	Yahoo-messages	30.86	3	9	230,863	47,886
*k* = 3 *l* = 4	SFHH-Conf	11.56	1	67	44,221	10,554
	As-Topology	2,185.33	9	159	16,068,985	12,6181
	Contacts-Dublin	17.49	2	120	192,318	18,141
	Enron-Email	1,527.17	8	30	40,484,380	1,350,257
	Digg-Friends	54.96	2	32	259,097	10,738
	Yahoo-messages	32.05	3	49	230,863	9,825
*k* = 3 *l* = 6	SFHH-Conf	12.63	1	97	44,221	8,032
	As-Topology	2,197.95	7	614	16,068,985	32,710
	Contacts-Dublin	19.10	2	224	192,318	10,259
	Enron-Email	1,527.50	8	30	40,484,380	1,350,257
	Digg-Friends	55.57	2	32	259,097	10,738
	Yahoo-messages	34.52	3	55	230,863	7,863
*k* = 4 *l* = 3	SFHH-Conf	9.98	1	62	87,189	5,932
	As-Topology	-	OOM	-	-	-
	Contacts-Dublin	21.16	2	74	192,318	41,959
	Enron-Email	-	OOM	-	-	-
	Digg-Friends	52.78	2	54	259,097	8,811
	Yahoo-messages	30.50	4	60	311,440	15,852
*k* = 4 *l* = 6	SFHH-Conf	191.19	4	3,097	87,189	5,932
	As-Topology	-	OOM	-	-	-
	Contacts-Dublin	86.88	4	6,603	192,318	1,666
	Enron-Email	-	OOM	-	-	-
	Digg-Friends	48.79	2	175	259,097	2,869
	Yahoo-messages	31.99	4	320	311,440	2,986
*k* = 4 *l* = 9	SFHH-Conf	378.52	4	10,916	87,189	2,623
	As-Topology	-	OOM	-	-	-
	Contacts-Dublin	118.13	4	38,389	192,318	357
	Enron-Email	-	OOM	-	-	-
	Digg-Friends	52.34	2	175	259,097	2,869
	Yahoo-messages	33.72	5	338	311,440	2,827
*k* = 5 *l* = 4	SFHH-Conf	96.65	4	446	31,228	31,228
	As-Topology	-	OOM	-	-	-
	Contacts-Dublin	52.19	3	575	192,318	13,404
	Enron-Email	-	OOM	-	-	-
	Digg-Friends	46.74	2	174	259,097	3,744
	Yahoo-messages	38.87	5	241	466,671	6,970
*k* = 5 *l* = 8	SFHH-Conf	-	OOM	-	-	-
	As-Topology	-	OOM	-	-	-
	Contacts-Dublin	632.27	7	530,099	192,318	119
	Enron-Email	-	OOM	-	-	-
	Digg-Friends	47.69	2	540	259,097	1,315
	Yahoo-messages	38.87	5	1,229	466,671	1,376
*k* = 5 *l* = 12	SFHH-Conf	-	OOM	-	-	-
	As-Topology	-	OOM	-	-	-
	Contacts-Dublin	-	OOM	-	-	-
	Enron-Email	-	OOM	-	-	-
	Digg-Friends	47.55	3	540	259,097	1,315
	Yahoo-messages	37.67	5	1,243	466,671	1,360

*For each experiment we report: (i) running time, (ii) maximum memory approximately used (GB), (iii) number of distinct motifs identified, (iv) number of occurrences of the most frequent motif, and (v) average number of motif occurrences. In the cases where no running time is reported, MODIT went out of memory (OOM) due to the high number of motifs present in the network*.

**Table 3:** Experiments on Dataset 1 with Δ = 3*r* and different combination of values of *k* and *l*.

**Configuration**	**Network**	**Time (*s*)**	**Mem (*GB*)**	**n**	**Max**	**AVG**
*k* = 3 *l* = 2	SFHH-Conf	3.91	<1	9	84,265	45,239
	As-Topology	80.61	6	10	16,068,985	1,988,007
	Contacts-Dublin	10.89	2	9	352,016	184,917
	Enron-Email	193.68	8	10	40,484,380	4,050,941
	Digg-Friends	54.84	2	9	540,758	69,030
	Yahoo-messages	33.02	3	10	389,792	59,811
*k* = 3 *l* = 4	SFHH-Conf	23.65	2	155	109,003	16,673
	As-Topology	2,191.53	9	202	16,068,985	106,737
	Contacts-Dublin	43.74	3	193	352,016	33,024
	Enron-Email	1,702.50	8	46	40,484,380	88,0880
	Digg-Friends	49.16	2	32	540,758	19,798
	Yahoo-messages	30.12	3	52	389,792	11,550
*k* = 3 *l* = 6	SFHH-Conf	49.99	3	488	109,003	9,248
	As-Topology	2,203.59	7	842	16,068,985	25,650
	Contacts-Dublin	69.95	3	1,035	352,016	8,690
	Enron-Email	1,719.70	8	56	40,484,380	723,600
	Digg-Friends	59.08	2	32	540,758	19,798
	Yahoo-messages	31.65	4	58	389,792	10,355
*k* = 4 *l* = 3	SFHH-Conf	23.00	2	79	282,531	50,403
	As-Topology	-	OOM	-	-	-
	Contacts-Dublin	42.01	3	82	352,016	88,450
	Enron-Email	-	OOM	-	-	-
	Digg-Friends	62.87	3	54	540,758	18,847
	Yahoo-messages	39.92	5	64	508,964	24,395
*k* = 4 *l* = 6	SFHH-Conf	-	OOM	-	-	-
	As-Topology	-	OOM	-	-	-
	Contacts-Dublin	894.01	7	13,421	352,016	5,487
	Enron-Email	-	OOM	-	-	-
	Digg-Friends	54.39	2	183	540,758	5,920
	Yahoo-messages	36.72	4	399	508,964	3,933
*k* = 4 *l* = 9	SFHH-Conf	-	OOM	-	-	-
	As-Topology	-	OOM	-	-	-
	Contacts-Dublin	-	OOM	-	-	-
	Enron-Email	-	OOM	-	-	-
	Digg-Friends	62.12	3	183	540,758	5,920
	Yahoo-messages	43.18	5	429	508,964	3,658
*k* = 5 *l* = 4	SFHH-Conf	340.22	6	633	1,607,533	90,145
	As-Topology	-	OOM	-	-	-
	Contacts-Dublin	165.53	4	672	352,016	39,229
	Enron-Email	-	OOM	-	-	-
	Digg-Friends	60.97	3	186	540,758	9,015
	Yahoo-messages	49.68	5	266	445,230	11,524
*k* = 5 *l* = 8	SFHH-Conf	-	OOM	-	-	-
	As-Topology	-	OOM	-	-	-
	Contacts-Dublin	-	OOM	-	-	-
	Enron-Email	-	OOM	-	-	-
	Digg-Friends	64.95	3	612	540,758	3,195
	Yahoo-messages	52.23	5	1,958	775,230	1,578
*k* = 5 *l* = 12	SFHH-Conf	-	OOM	-	-	-
	As-Topology	-	OOM	-	-	-
	Contacts-Dublin	-	OOM	-	-	-
	Enron-Email	-	OOM	-	-	-
	Digg-Friends	75.33	4	612	540,758	3,195
	Yahoo-messages	55.36	5	2,016	775,230	1,533

*For each experiment we report: (i) running time, (ii) maximum memory approximately used (GB), (iii) number of distinct motifs identified, (iv) number of occurrences of the most frequent motif, and (v) average number of motif occurrences. In the cases where no running time is reported, MODIT went out of memory (OOM) due to the high number of motifs present in the network*.

**Table 4:** Experiments on Dataset 1 with Δ = 3*r* and different combination of values of *k* and *l*.

**Configuration**	**Network**	**Time (*s*)**	**Mem (*GB*)**	**n**	**Max**	**AVG**
*k* = 3 *l* = 2	SFHH-Conf	3.76	<1	9	124,450	62,716
	As-Topology	84.85	6	10	16,068,985	2,056,209
	Contacts-Dublin	12.94	2	9	497,191	253,808
	Enron-Email	192.64	8	10	40,484,380	4,051,491
	Digg-Friends	47.80	3	9	791,420	97,274
	Yahoo-messages	33.82	4	10	510,303	73,198
*k* = 3 *l* = 4	SFHH-Conf	53.57	1	198	222,458	32,430
	As-Topology	2,277.25	8	209	16,068,985	108,927
	Contacts-Dublin	85.07	3	203	497,191	67,993
	Enron-Email	1,716.09	8	54	40,484,380	750,629
	Digg-Friends	58.37	2	33	791,420	26,977
	Yahoo-messages	32.04	4	55	510,303	13,368
*k* = 3 *l* = 6	SFHH-Conf	213.16	4	1,044	222,458	19,003
	As-Topology	2,282.17	7	1,026	16,068,985	22,251
	Contacts-Dublin	235.862	4	1,652	497,191	17,978
	Enron-Email	1,752.45	8	95	40,484,380	426,725
	Digg-Friends	59.23	3	33	791,420	26,977
	Yahoo-messages	33.52	4	61	510,303	12,053
*k* = 4 *l* = 3	SFHH-Conf	38.82	3	82	578,085	90,761
	As-Topology	-	OOM	-	-	-
	Contacts-Dublin	70.91	4	82	634,058	156,582
	Enron-Email	-	OOM	-	-	-
	Digg-Friends	67.76	3	56	791,420	29,108
	Yahoo-messages	43.18	5	64	890,576	33,600
*k* = 4 *l* = 6	SFHH-Conf	-	OOM	-	-	-
	As-Topology	-	OOM	-	-	-
	Contacts-Dublin	-	OOM	-	-	-
	Enron-Email	-	OOM	-	-	-
	Digg-Friends	56.39	3	201	791,420	8,659
	Yahoo-messages	40.62	5	430	890,576	5.030
*k* = 4 *l* = 9	SFHH-Conf	-	OOM	-	-	-
	As-Topology	-	OOM	-	-	-
	Contacts-Dublin	-	OOM	-	-	-
	Enron-Email	-	OOM	-	-	-
	Digg-Friends	58.64	3	201	791,420	8,659
	Yahoo-messages	50.21	2	460	890,576	4,702
*k* = 5 *l* = 4	SFHH-Conf	1,001.28	7	677	4,526,786	217,632
	As-Topology	-	OOM	-	-	-
	Contacts-Dublin	391.67	6	682	806,886	90,608
	Enron-Email	-	OOM	-	-	-
	Digg-Friends	77.35	4	203	791,420	15,624
	Yahoo-messages	63.14	5	276	1,346,934	14,226
*k* = 5 *l* = 8	SFHH-Conf	-	OOM	-	-	-
	As-Topology	-	OOM	-	-	-
	Contacts-Dublin	-	OOM	-	-	-
	Enron-Email	-	OOM	-	-	-
	Digg-Friends	83.79	4	737	791,420	5,228
	Yahoo-messages	64.45	5	2,258	1,346,934	2,125
*k* = 5 *l* = 12	SFHH-Conf	-	OOM	-	-	-
	As-Topology	-	OOM	-	-	-
	Contacts-Dublin	-	OOM	-	-	-
	Enron-Email	-	OOM	-	-	-
	Digg-Friends	83.96	4	737	791,420	5,228
	Yahoo-messages	71.64	5	2,325	1,346,934	2,064

*For each experiment we report: (i) running time, (ii) maximum memory approximately used (GB), (iii) number of distinct motifs identified, (iv) number of occurrences of the most frequent motif, and (v) average number of motif occurrences. In the cases where no running time is reported, MODIT went out of memory (OOM) due to the high number of motifs present in the network*.

Interestingly, in some networks (e.g., Enron-Email, Digg-Friends) we observe that keeping Δ and *k* fixed and varying *l*, the number of distinct motifs, the number of occurrences of the most frequent motif and average number of occurrences remain the same.

### 3.2. Comparison With Paranjape's Algorithm

Finally we compared MODIT with the algorithm proposed by Paranjape et al. (2017) on the networks of Dataset 2.

For the comparison, we set *k* = 3 and *l* = 3 because Paranjape's algorithm can handle only motifs with 2 or 3 nodes and 3 edges.

Results are reported in Table 5 and show that Paranjape's algorithm is much faster than MODIT. This gap is mainly due to the fact that Paranjape's method uses a series of efficient dynamic programming algorithms, which are specifically designed to count specific classes of motifs, i.e., motifs with 3 edges. On the other hand, MODIT is general and designed to find motifs of any size and any type. Furthermore, Paranjape's algorithm searches only motifs having exactly the specified number of nodes and edges. On the other hand, MODIT looks for all motifs having at most the number of nodes and edges specified by the user.

**Table 5:** Comparison between MODIT and Paranjape's algorithm.

**Network**	**Δ**	**Paranjape et al**.	**MODIT**
CollegeMsg	350,000	0.18	1,148.96
Email-Eu-core-temporal-Dept1	70,000	0.11	81.01
Email-Eu-core-temporal-Dept2	70,000	0.09	56.71
Email-Eu-core-temporal-Dept3	70,000	0.04	3.47
Email-Eu-core-temporal-Dept4	70,000	0.08	46.45
Email-Eu-core-temporal	70,000	0.66	281.32

*For each network, we indicate the value of Δ used and the running time (in seconds) of both algorithms. MODIT was run with k = 3 and l = 3 because Paranjape's algorithm can only handle motifs with 2 or 3 nodes and 3 edges*.

## 4. Conclusions

In this paper, we presented MODIT (MOtif DIscovery in Temporal Networks), an algorithm for counting motifs of any size in temporal networks, inspired by a very recent algorithm for subgraph isomorphism in temporal networks, called TemporalRI. Given the three parameters *k*, *l*, and Δ, MODIT scans the whole temporal graph to search for all subgraphs having at most *k* nodes and *l* edges and in which the difference between the maximum and the minimum timestamp is no greater than Δ. We ran MODIT on a dataset of real temporal networks of medium and large size by varying Δ, the maximum number of nodes and edges. We also compared MODIT with the algorithm proposed by Paranjape et al. (2017) using a different dataset of temporal networks downloaded from SNAP.

For the future, we plan to:

1. Introduce measures of statistical significance of motifs similar to the ones already devised for static graphs (i.e., the *z*-score);
2. Make MODIT iterative, in order to reduce the overhead introduced by recursion, and optimize the search strategy;
3. Implement MODIT on a SPARK framework, to manage very large networks.

## Data Availability Statement

The original contributions presented in the study are included in the article/supplementary material, further inquiries can be directed to the corresponding author/s. The source code of MODIT can be found at the following URL: https://github.com/RobertoGrasso96/MODIT.

## Author Contributions

AP and GM coordinated the project. GM and RG developed the algorithm. RG performed the experiments. RG, GM, AF, and AP wrote the manuscript. All authors conceived the project, read, and approved the final manuscript.

## Funding

AP and AF have been partially supported by the following research projects: MIUR PON BILIGeCT Liquid Biopsies for Cancer Clinical Management (CUP B96G18000590005); PO-FESR Sicilia 2014-2020 DiOncoGen: Innovative diagnostics (CUP G89J18000700007).

## Conflict of Interest

The authors declare that the research was conducted in the absence of any commercial or financial relationships that could be construed as a potential conflict of interest.

## Publisher's Note

All claims expressed in this article are solely those of the authors and do not necessarily represent those of their affiliated organizations, or those of the publisher, the editors and the reviewers. Any product that may be evaluated in this article, or claim that may be made by its manufacturer, is not guaranteed or endorsed by the publisher.

## Footnotes

^1^ https://snap.stanford.edu/data/index.html
